# A Clinical‐Scale Microfluidic Respiratory Assist Device with 3D Branching Vascular Networks

**DOI:** 10.1002/advs.202207455

**Published:** 2023-04-24

**Authors:** Brett C. Isenberg, Else M. Vedula, Jose Santos, Diana J. Lewis, Teryn R. Roberts, George Harea, David Sutherland, Beau Landis, Samuel Blumenstiel, Joseph Urban, Daniel Lang, Bryan Teece, WeiXuan Lai, Rose Keating, Diana Chiang, Andriy I. Batchinsky, Jeffrey T. Borenstein

**Affiliations:** ^1^ Bioengineering Division Draper Cambridge MA 02139 USA; ^2^ Autonomous Reanimation and Evacuation (AREVA) Research Program The Geneva Foundation San Antonio TX 78234 USA

**Keywords:** 3D, microfluidics, hemocompatibility, ECMO, respiratory assist, oxygenation, vascular

## Abstract

Recent global events such as COVID‐19 pandemic amid rising rates of chronic lung diseases highlight the need for safer, simpler, and more available treatments for respiratory failure, with increasing interest in extracorporeal membrane oxygenation (ECMO). A key factor limiting use of this technology is the complexity of the blood circuit, resulting in clotting and bleeding and necessitating treatment in specialized care centers. Microfluidic oxygenators represent a promising potential solution, but have not reached the scale or performance required for comparison with conventional hollow fiber membrane oxygenators (HFMOs). Here the development and demonstration of the first microfluidic respiratory assist device at a clinical scale is reported, demonstrating efficient oxygen transfer at blood flow rates of 750 mL min⁻^1^, the highest ever reported for a microfluidic device. The central innovation of this technology is a fully 3D branching network of blood channels mimicking key features of the physiological microcirculation by avoiding anomalous blood flows that lead to thrombus formation and blood damage in conventional oxygenators. Low, stable blood pressure drop, low hemolysis, and consistent oxygen transfer, in 24‐hour pilot large animal experiments are demonstrated – a key step toward translation of this technology to the clinic for treatment of a range of lung diseases.

## Graphical abstract

The first microfluidic oxygenator demonstrated at clinical scale is reported, utilizing bioinspired design principles to achieve high efficiency oxygen transfer and excellent blood pressure stability in 24‐h testing in large animals. This technology has the potential to enable safer, simpler, and more accessible extracorporeal membrane oxygenation (ECMO), addressing urgently needed advances in the treatment of respiratory failure.

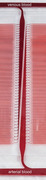

## 1 Introduction

Lung diseases represent the third leading cause of death in the United States behind heart disease and cancer,^[^ ^1^ ^]^ and recent events, including the COVID‐19 pandemic, have further exacerbated this situation, with high morbidity and mortality resulting from acute respiratory distress syndrome.^[^ ^2^ ^]^ The predominant treatment for acute lung injury is invasive mechanical ventilation,^[^ ^3^ ^]^ which is highly efficacious in addressing hypoxemia and hypercarbia but is associated with complications including barotrauma arising from high pressures from the ventilator, biotrauma stemming from exposure of the lungs to highly concentrated oxygen flows, and an elevated risk of infection resulting from microbial colonization in the ventilator lines.^[^ ^4^, ^5^ ^]^ These safety risks have spurred interest in approaches that permit injured or diseased lungs to rest while treating hypoxemia, principally through the use of extracorporeal membrane oxygenation (ECMO) which involves placement of central venous and arterial catheters and circulation of blood outside the body and through a hollow fiber membrane oxygenator (HFMO),^[^ ^6^ ^]^ within which oxygenation and CO_2_ removal takes place. First explored by Dr. Robert Bartlett as a treatment for newborns with meconium aspiration syndrome in 1975,^[^ ^7^ ^]^ significant clinical and technological advances continued through the 1980s and 1990s.^[^ ^8^, ^9^, ^10^, ^11^ ^]^ ECMO has gained ascendancy in adult populations after initial demonstration of a survival benefit in the CESAR trial,^[^ ^12^ ^]^ and following the 2009 H1N1^[^ ^13^ ^]^ and COVID‐19 pandemics.^[^ ^14^, ^15^ ^]^ In spite of rapid progress,^[^ ^16^, ^17^ ^]^ ECMO generally remains a choice of last resort,^[^ ^11^, ^18^ ^]^ due to factors including a requirement for a central vascular access, the need for a large team of highly trained specialists managing the patient and circuit, and complex administration protocols for anticoagulants to prevent thrombus formation.^[^ ^19^, ^20^, ^21^ ^]^ Therefore ECMO procedures represent only a fraction of the total patient population suffering from respiratory failure, most of whom still receive invasive mechanical ventilation.^[^ ^22^ ^]^

Limitations preventing expanded use of ECMO arise principally from the complexity of the blood circuit, which leads to complications such as clotting, bleeding, and inflammatory responses.^[^ ^23^, ^24^, ^25^, ^26^ ^]^ Beyond systemic anticoagulation, most efforts to further address these complications are directed toward mitigating surface‐induced activation of the blood, via application of anti‐thrombotic surface treatments applied to the HFMO cartridge and to associated tubing and other circuit elements.^[^ ^27^, ^28^, ^29^ ^]^ However, another major cause of hemocompatibility issues in ECMO stems from the presence of non‐physiologic blood flows in the circuit^[^ ^30^, ^31^, ^32^, ^33^ ^]^: regions of excessive shear stress, zones of blood stasis, and transition regions, all serving as a nidus for clot formation. The fundamental geometry of HFMO cartridges, in which blood flows through large bore plastic tubes and over oxygen‐carrying fiber mats within a large housing, limits the ability to precisely control blood flow circulation patterns or shear rates on a local level.^[^ ^20^, ^32^, ^34^ ^]^ It is important to note that the cartridge is far from the only source of blood circuit complications, as extensive efforts have been devoted to the development of oxygenator pumps and other circuit elements that are known to be responsible for blood damage and clotting. In the field of microfluidic oxygenators, promising advances in the demonstration of pumpless configurations have been reported, toward addressing these concerns.^[^ ^35^ ^]^

One of the most promising routes of innovation in ECMO entails the use of microfluidics technology for the design and construction of the oxygenator, effectively replacing the HFMO configuration with a branching network of microchannels that recapitulates key aspects of the lung vasculature.^[^ ^36^, ^37^ ^]^ A central rationale for introducing a microfluidics‐based architecture is that it enables devices with thinner gas transfer membranes and shallower blood channels that are possible with HFMO devices, reducing diffusion distances and hence increasing the efficiency of gas transfer while reducing membrane surface area and blood prime volume.^[^ ^38^, ^39^, ^40^, ^41^, ^42^ ^]^ This has been demonstrated in a large number of recently reported studies, where high oxygen transfer efficiencies were obtained in small‐scale prototype devices tested on the bench^[^ ^38^, ^39^, ^43^ ^]^ and, in selected cases, in short‐term experiments in small animal models.^[^ ^35^, ^44^, ^45^ ^]^ However, in spite of the promise of microfluidic oxygenators and the encouraging test data obtained from gas transfer studies, these devices have largely been limited to very low blood flow rates.^[^ ^46^ ^]^ Furthermore, early results from small animal experiments do not indicate that thrombus formation or other blood circuit complications are mitigated by these microfluidic designs.^[^ ^35^, ^44^ ^]^ Microfluidic design features alone do not necessarily address a key limitation of the HFMO: the presence of non‐physiological flow patterns of blood flow. The high surface‐to‐volume ratio for shallow channel devices may be another impediment. Virtually all microfluidic oxygenators reported in the literature are constructed using photolithographically‐based microfabrication techniques, where inverse patterns of rectangular channel networks are created on silicon wafers,^[^ ^44^ ^]^ followed by microreplication with silicone films to create networks of microchannels. Photolithographic processes generally patterned features all at a single height, resulting in a constant channel depth in the replica‐molded cast. This imposes a severe limitation on the design that renders the resulting blood flow networks vastly different from the true branching nature of the physiological microcirculation, necessitating extremely wide trunk lines that are as shallow as the narrowest capillary‐like channels. These devices have proven very difficult to scale beyond 10 mL min⁻^1^ in a single layer, and, therefore, stacks of as many as 16 layers have been required to reach blood flows of 100 mL min⁻^1^,^[^ ^35^ ^]^ with vertical pipes in distribution manifolds connecting the layers. These vertically oriented manifolds are inherently thrombogenic because they comprise sharp, rectangular junctions between the vertical trunk line and the lateral feed channels entering each layer. Animal studies of these devices have been limited to durations of 2–3.5 h, with heavy thrombus formation observed unless coagulation mitigation procedures such as heparin coatings and nitric oxide administration are invoked.^[^ ^35^, ^44^ ^]^ We propose that these difficulties in blood flow dynamics originate from the non‐physiologic nature of the branching networks in these devices, both within layers and between layers, and that a more bioinspired branching architecture is potentially capable of significantly reducing shear stress and hemolysis, as demonstrated by very low levels of plasma‐free hemoglobin (PfHb) during extended testing.

Here we present the first significant scaling of a microfluidic blood oxygenators (BLOx) built using fully 3D branching networks of microchannels designed on the basis of principles drawn from the in vivo microcirculation. These principles include bounding the shear rates across the entire network within ranges known to avoid tissue factor‐induced activation and platelet activation, targeting shear stress levels near 3.5 Pa^[^ ^47^, ^48^, ^49^ ^]^ in the large trunk lines as well as the smallest gas transfer channels. In addition, Murray's Law,^[^ ^50^ ^]^ a design paradigm first reported nearly 100 years ago to describe the way that branched daughter channel diameters relate to the diameter of the parent vessel, is used to guide branches and bifurcations across these networks.^[^ ^51^ ^]^ These designs are realized in the construction process by invoking 3D high precision micromilling of master molds used for silicone microreplication, a process that enables smooth and gradual transitions from deep to shallow and narrow to wide channels. This approach is used to fashion both the in‐network channel patterns and the blood distribution manifolds that join stacks of layers. Resulting BLOx devices are tested in vitro for blood oxygen transfer, demonstrating very high oxygen transfer efficiencies consistent with computationally modeled performance predictions. We then present the first large animal studies conducted with a microfluidic oxygenator, assessing the feasibility and performance (evaluated by the stability of the blood flow rate, blood pressure drop, and oxygen transfer rate), and one important metric of blood health (testing for the presence of hemolysis by monitoring PfH), of a group of three devices operated at 750 mL min⁻^1^ blood flow in pilot studies carried to 24 h in a porcine model. Importantly, unlike prior short‐term microfluidic oxygenator animal studies, these studies were conducted with microfluidic devices that received no anti‐thrombogenic surface coatings or nitric oxide administration, only systemic introduction of heparin to maintain activated clotting time (ACT) levels of 250–350 s. We demonstrate low PfHb levels‐ a key metric of shear stress, over the entire 24‐h duration of circulation. These groundbreaking results in terms of scaling toward clinical blood flows represents an important step forward toward the development and demonstration of microfluidic oxygenators as a credible alternative to HFMO cartridges for safer, simpler, and more widely available ECMO for critical care patients.

## 2 Results

### 2.1 Iterative Modeling‐Yielded Multi‐Banked Channel Design

**Figure 1:**
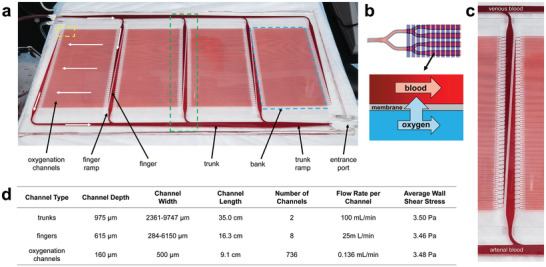
Overview of single layer of the BLOx device. a) An image of a BLOx layer showing the key blood‐side features and nomenclature used. White arrows show the general path of the blood through one part of the network. b) A magnified view of the dashed yellow region of Panel a showing overlap between the blood (red) and oxygen (blue) channels as well as a schematic of the oxygen transfer across the membrane in the overlap region. c) A magnified view of the dashed green region of Panel a showing greater detail of smoothly transitioning 3D distribution network that maintains the wall shear stress within a narrow range for the goal of maintaining good blood health. Darker, deoxygenated venous blood enters from the top trunk and bright, oxygenated arterial blood leaves the oxygenation channels to the bottom trunk. d) A table of channel dimensions, flow rates, and shear stresses for individual channel types in the single layer BLOx device. The ranges shown for the trunk and finger channel types indicate their narrowest to widest values as they taper from one end to the other.

Computational modeling described in the Methods section was used to generate a 4‐bank design, illustrated and annotated in **Figure** **1**, that was the most compatible with a bifurcating manifold network. Here we present the overall structure and detailed features of this design. The final 4‐bank design contained 736 individual oxygenation channels, each of which were 9.1 cm long, 500 µm wide, and 160 µm deep. Within each bank, oxygenation channels are connected upstream and downstream by a two‐level bifurcating channel network to large distribution channels referred to as “fingers”, that connect with an even larger distribution channel referred to as a “trunk”. Trunk lines interface directly with the entrance and exit ports of the layer. At each convergence point or change in channel dimensions, carefully engineered transitions regions allow flow to be maintained in a narrow range of shear stress and to evenly distribute blood across all channels (Figures S2,S3,S4,S5, Supporting Information). Layer prime volume is 14.7 mL with a residence time of 8.8 s at a blood flow rate of 100 mL min⁻^1^. Final layer dimensions are 253 mm wide by 478 mm long.

The primary design goal of the oxygen layer was to ensure uniform oxygen distribution across the network to provide optimum oxygen transfer at all points. Unlike the blood side, the oxygen side is comprised of a single large bank of parallel channels that run perpendicular to the blood channels and connect to the entrance/exit ports via large trunk lines. This arrangement allows for a balance between oxygen transfer area and support for the membrane, which is prone to bowing at higher oxygen pressures. The oxygen channels are 500 µm wide, 300 µm deep, and 17.2 cm long, resulting in a total overlap transfer area with the blood side of 0.0167 m^2^ (total blood contacting surface area is significantly higher, given the relatively large area where blood channels overlap bulk silicone rather than thin membrane regions.) The oxygen layer footprint is the same as for the blood side.

### 2.2 BLOx Construction

**Figure 2:**
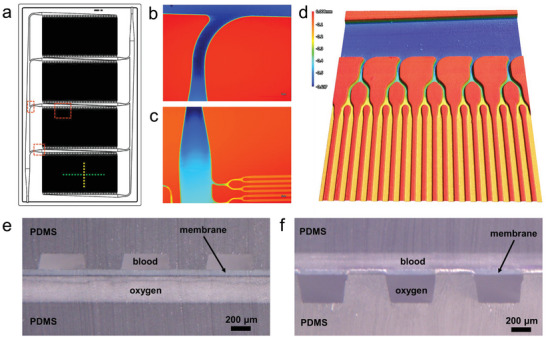
Images of a fabricated BLOx layer. a) Computer‐aided design (CAD) layout of the BLOx device showing the 4‐bank configuration with the blood inlet at the lower left and the blood outlet at the upper right. The dashed lines and boxes indicate regions of interest that are shown in greater detail in the remaining panels. b) – d) metrology images of trunk, finger, and channel regions called out in Panel a. Images were obtained with laboratory microscope capable of optical profilometry scanning to determine channel depth as it varies across the vascular network pattern in a layer. b) Branch point in the in‐layer manifold with curved transition, shown visually and with a false color map to indicate the channel depth, showing the deeper horizontal channel (dark blue) in the trunk line leading to a shallower vertical finger ramp channel (lighter blue). c) False color map showing the entry point (finger ramp) as the deepest channel (dark blue), moving into lighter blue along the vertical finger channel, and branching into the shallowest lateral oxygenation channels (shown in yellow and orange.) d) 3D close‐up of lateral oxygen transfer channels (orange) coming from steadily deeper bifurcation points (yellow) and tracing back to the trunk line (dark blue.) The color scale bar spans ≈0.6 mm. e)‐f) Light micrographs of cross‐sections of a single layer along the dashed green (e) and dashed yellow (f) lines in Panel a showing the blood and oxygen channels separated by a thin, gas permeable membrane. In Panel e, the blood channels run perpendicular to the plane of the image and the oxygen channels run parallel, whereas the opposite is shown in Panel f.

3D precision micromilling and silicone microreplica molding resulted in accurate replication of the smooth transitions designed into the two‐level bifurcating channel network of the BLOx vascular layer, as verified by dimensional and surface characterization described in the following. **Figure** **2** summarizes the surface profile of a molded vascular layer. Three regions of the 4‐bank vascular design shown in Figure 2a were measured using an optical profilometer. The color gradients demonstrate the smoothly transitioning regions of channel geometries in major branching sections of the layer.

### 2.3 In Vitro Testing

#### 2.3.1 Single‐Layer Microfluidic Oxygenator Performance

**Figure 3:**
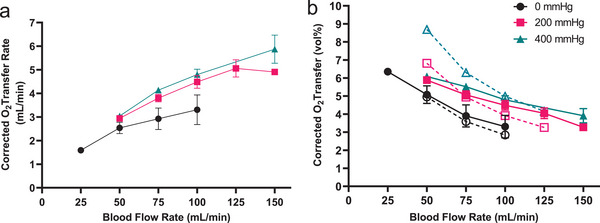
Transfer of oxygen into the blood channel of the device was measured over a range of flow rates and compared to predicted values. a) The corrected oxygen transfer rate increased with increased blood flow and with increased applied back pressure, reaching over 5 mL O2/min at the maximum conditions tested. b) At a nominal flow rate of 100 mL min⁻^1^, the volume of oxygen transferred into a unit volume of blood (vol%) reaches 3.3 vol% and 4.8 vol% with 0 and 400 mm Hg applied O2 back pressure, respectively. N = 2–7 replicates for each data point. Dotted lines and open symbols represent predicted transfer of oxygen based on simulation results.

Single layer device performance was evaluated by quantifying the transfer of oxygen into the blood channel over a range of blood flow rates, as described in Experimental Section, and in previous reports.^[^ ^43^, ^45^, ^52^ ^]^ Blood oxygenation levels were measured at discrete flow rates ranging from 25 mL min⁻^1^ to 150 mL min⁻^1^ and were compared to predicted results from computational modeling. Additionally, experiments were conducted across a range of oxygen back pressure levels applied to the outlet of the oxygen chamber, as described in Experimental Section, operating under the concept that oxygen at higher pressure is at a higher concentration which should increase the concentration gradient across the membrane and resulting in an increased rate of diffusion. **Figure** **3** demonstrates the corrected oxygen transfer rate in single layer BLOx devices in both mL O_2_/min (Figure 3a) and in volume percent (vol%, defined as the volumetric O_2_ transfer rate normalized by the blood flow rate, see Supporting Information 1) (Figure 3b). The average oxygen transfer rate was 3.3 vol% at 100 mL min⁻^1^ of blood flow through the device, a level of transfer that corresponds to raising oxygen saturation from 75% – 95%.^[^ ^53^ ^]^ This result was obtained with zero oxygen back pressure applied to the outlet of the oxygen chamber. Increasing the oxygen back pressure to 400 mm Hg resulted in an oxygen transfer rate of 4.8 vol%. As reported elsewhere,^[^ ^43^ ^]^ a transfer rate of 4.95 vol% would correspond to raising the oxygen saturation from 65–95%, and therefore this performance very closely approaches that level. Maintaining the 400 mm Hg back pressure and increasing blood flow to 150 mL min⁻^1^ in a single layer device resulted in an average oxygen transfer rate of just under 4 vol%, highlighting the significant impact of applied back pressure on overall oxygen transfer.

The measured performance matched closely with predicted oxygen transfer rates (Figure 3b, dotted lines), particularly at a blood flow rate of 100 mL min⁻^1^, where measured transfer rates were within 4–16% of predicted values depending on applied back pressure conditions. The discrepancy between measured and predicted values was higher at the ends of the tested blood flow range. As can be seen in Figure 3b, the computational model was far more predictive across the range of blood flow rates tested in cases with zero applied oxygen back pressure, while it diverged significantly from the experimental data at low blood flow rates for the higher back pressures.

**Figure 4:**
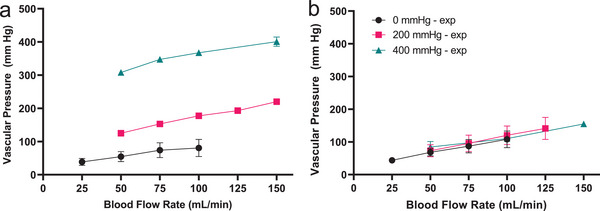
Pressure recorded in the blood layer depends on flow rate, applied oxygen back pressure, and oxygen layer design. a) The pressure drop remains below 100 mmHg when there is no additional back pressure applied to the oxygen channel. In the nominal design of the oxygenator, the blood pressure drop increases with added back pressure. b) modifications to the oxygen layer design eliminates membrane bow in fingers and trunk regions of the device, collapsing pressure drop in the blood channel across applied back pressures.

Pressure in the blood layer was monitored during oxygen transfer tests at each blood flow rate and applied oxygen back pressure. At 100 mL min⁻^1^ blood flow, the measured pressure drop was ≈80 mm Hg. Increasing applied oxygen back pressure increased the pressure drop measured in the blood channel by 4.5x, a result we hypothesized was due to membrane bow into the blood channels in the on‐layer blood distribution manifolds. To address this, a preliminary design modification comprising additional mechanical membrane supports was incorporated into BLOx (see Supporting Information 5) to mitigate high pressures recorded in **Figure** **4**a. Data in Figure 4b illustrates the significant reduction of blood pressure in the modified design, where at 100 mL min⁻^1^ blood flow, the average pressure did not exceed 105 mm Hg, regardless of applied oxygen back pressure.

### 2.4 In Vivo Animal Testing

#### 2.4.1 8‐Layer Microfluidic Oxygenator Performance

**Figure 5:**
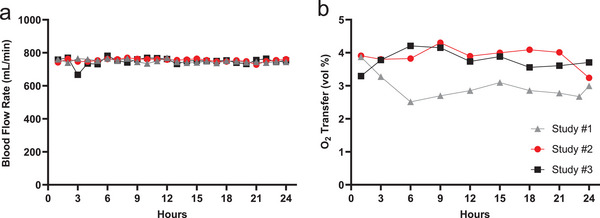
Performance of three microfluidic oxygenator devices was evaluated over 24 h in animal studies. a) The flow rate maintained a target of 750 mL min⁻^1^ for 24 h. b) Oxygen transfer in study #1 was ≈3 vol% compared to ≈4 vol% in studies #2 and #3, each relatively stable for 24 h, indicating consistent performance of the device over time. All animals survived and there were no untoward safety effects during these pilot studies.

The three 8‐layer BLOx devices were evaluated at clinically relevant blood flow rates potentially applicable for neonatal, pediatric, or adult partial lung support of 750 mL min⁻^1^ in a 24‐h porcine feasibility and safety study in swine. Briefly, all animals were kept in an intensive care unit in supine position and were supported by intravenous anesthesia, continuous fluid administration, and mechanical ventilation as previously described by our group.^[^ ^54^ ^]^ After initial lineup the microfluidic device was connected to the animals via a dual lumen veno‐venous catheter inserted into the jugular vein. Systemic anticoagulation was achieved by continuous administration of unfractionated heparin.^[^ ^54^ ^]^ Due to the observation that the application of oxygen back pressure increased the blood pressure drop significantly in the original designs for the oxygen channel networks as mentioned above, these animal studies were conducted with zero applied back pressure. As shown in **Figure** **5**, all three BLOx devices maintained consistent performance over 24 h duration of the study, in terms of blood flow rate, blood pressure drop, and oxygen transfer rate. As shown in Figure 5a, these devices supported the target blood flow rate of 750 mL min⁻^1^ with a standard deviation of 14.8 mL min⁻^1^ across the entire 24 h test in each of the three studies. Figure 5b shows that the measured performance in terms of oxygen transfer matched or exceeded the predicted oxygen transfer of 3 vol% with no applied oxygen back pressure. The corrected oxygen transfer rate in the first study averaged near 3.0 vol% and was 3.9 and 3.8 vol% in studies 2 and 3, respectively.

**Figure 6:**
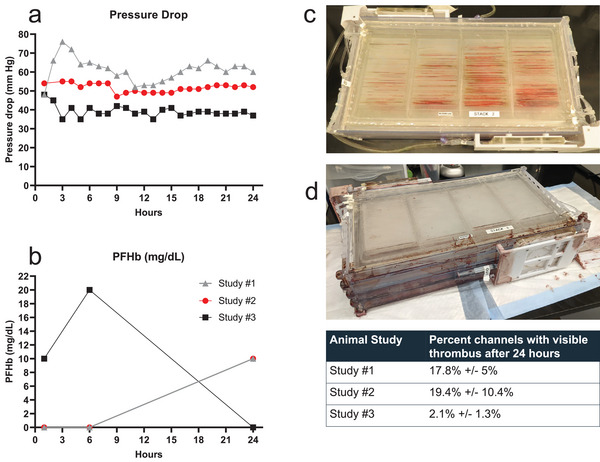
Readouts indicative of blood flow conditions and hemolysis were measured over 24 h for each of the three animal studies. a) Pressure drop was measured in the vascular channel. b) Plasma‐free hemoglobin (PfHb) was measured in blood sampled immediately post‐device exit. c–d) Photographs of 8‐layer devices following 24‐h Animal Study #2 and Animal Study #3 after gentle rinsing to remove whole blood from channels. (Table) Remaining visible clots were analyzed using a laboratory camera to count the number of microchannels in which one or more thrombi were present; ≈17–19% of the channels (assessed across each of the 8 layers) showed the presence of at least one visible thrombus in Studies #1 and # 2. An average of 2.1% of channels had at least one visible thrombi in the device tested in Animal Study #3. Note that the pressure drop across the device was not impacted by the presence of these thrombi. The device from Animal study #3 did incur a small leak in a manifold region during the testing that did not impact oxygenation, pressure drop, or thrombus formation, as each of these parameters was similar to observed performance of the device for Animal Study #2.

Pressure drop across the blood channel network was also evaluated in the animal study. In each of the three studies, the pressure drop remained relatively stable for 24 h, with variability in some studies but no perceptible rise in pressure across the experiment, as seen in **Figure** **6**a. Animal study #1 had a brief spike of blood pressure drop seen at the 3‐h mark, before steadying out ≈60 mmHg for the remainder of the study. Studies #2 and #3 were relatively stable ≈50 and 40 mmHg, respectively for 24 h. PfHb (Figure 6b) measured at the device exit did not become significantly elevated to a degree that would indicate a clinical concern of hemolysis during mechanical circulatory support (clinical diagnostic threshold >30–50 mg dL⁻^1^).^[^ ^55^ ^]^ This indicates that blood circulation through the BLOx device did not elicit significant injury to red blood cells that would be detrimental. Additional analyses addressing clinical course, systemic effects including the end‐of study histological evaluation are currently ongoing and will be reported elsewhere. Of note, however, no untoward effects were observed during the 24‐h experiment and all animals survived until the end of the study. At the conclusion of the 24‐h studies, devices were rinsed to assess residual clotting, as a means to assess the propensity for microchannel blocking even in the presence of systemic heparinization. In spite of the fact that no surfaces in this microfluidic oxygenator were functionalized with anti‐thrombotic coatings, after rinsing there were low to moderate levels of thrombi present in the channels (Figure 6c,d). ≈17–19% of channels showed evidence of thrombus formation in devices evaluated in animal studies #1 and #2, but as pressure monitoring shows, the thrombi that were present in the devices did not contribute to a detectable increase in pressure drop across the oxygenator. The stack evaluated in Animal Study #3 had perceptibly lower number of visible thrombi at ≈2% of oxygenation channels. The lower percent of channel blockages in Study #3 matches the trends across studies seen in the pressure drop and platelet count, where the magnitude of each was lower in the third study compared to the first two studies. These trends are being further explored in ongoing and future planned studies with larger statistics.

## 3 Discussion

The microfluidic oxygenator described here is the largest scale microfluidic oxygenator ever reported, and may represent the highest flow microfluidic device ever constructed. The blood flow rate of 750 mL min⁻^1^ renders this device the first clinical‐scale microfluidic oxygenator, equivalent to the lower end of the blood flow rate of a neonatal oxygenator, and therefore enables future studies to compare the performance of a microfluidic design to an HFMO in terms of gas transfer and hemocompatibility. The microfluidic design reported here is motivated by the opportunity to address limitations of traditional HFMO in terms of blood health, by recapitulating key aspects of the microcirculation such as vessel dimensions designed for optimum oxygen transfer and minimal pressure drop as well as flow patterns that allow for smooth transitions between large high‐flow distribution channels and the small, low‐flow channels where oxygen transfer primarily occurs.^[^ ^56^ ^]^ While considerable progress has already been made in advancing microfluidic ECMO toward clinical relevance, three areas have been particularly lacking in the field: tight control of shear stress at all points in the network, scalable operation at clinically relevant flow rates, and stable long‐term performance. We believe that the data presented here represent a major step forward on all three fronts and demonstrate a clear path to further improved performance.

The highest blood flow rate for a microfluidic ECMO device prior to this work reported 5 vol% oxygen transfer at 240 mL min⁻^1^ using an 8‐layer device^[^ ^52^ ^]^ based on a previously reported design,^[^ ^57^ ^]^ but this device exhibited high blood pressure drop and was not tested in an animal model. The highest blood flow rate in a microfluidic oxygenator tested in an animal model was reported by Dabaghi et al., where devices achieved 95% oxygen saturation at a flow rate under 100 mL min⁻^1^ in a 16‐layer stack,^[^ ^35^ ^]^ a configuration that would require 167 layers to reach a neonatal oxygenator scale (a comparison of the performance metrics from two recent animal studies using microfluidic oxygenators with those from the current study is summarized in **Table** **1**). In the table, we provide a synopsis of the animal study parameters as well as key device parameters including the number of layers and the active membrane surface area (area of blood channels overlapping oxygen channels). As the table indicates, we have expanded our throughput to 750 mL min⁻^1^ by increasing the flow rate of our individual layers by fourfold, while significantly reducing the pressure drop across each layer, enabling a neonatal device scale with an 8‐layer device. We also compare device performance to the Getinge Quadrox‐I oxygenator based on two clinical references in the literature.^[^ ^58^, ^59^ ^]^

**Table 1:** Performance comparison to recently published in vivo microfluidic ECMO devices

Device	Device flow rate [mL/min]	Layers per device	Sweep gas	Vol% ^a)^	Pressure drop [mmHg]	Transfer area exchange efficiency ^a)^ [mL/min/m^2^]	Residence Time [s]	Animal Model	Hct [%]	Duration [hr]
Current work	750	8	100% O_2_, 1–3 L/min	2.9% (3.4%)	51	164 (192)	9	Adult Yorkshire pig	27‐29	24
Dabaghi^[^ ^35^ ^]^	67	16	100% O_2_, passive	2.8% (6.0%)	48	31 (67)	5	Newborn piglet	23‐27	3.3
Thompson^[^ ^44^ ^]^	12	1	100% O_2_, 0.034 L/min	2.9% (3.7%)	66	109 (141)	1.7	adult New Zealand white rabbit	21	2
Getinge Quadrox‐i Neonatal^[^ ^59^ ^]^	1400	NA	80% O_2_, 1.6 L/min	4.4% (NA)	58	164 (NA)	1.7	Human	> = 30	1.4^b)^
Getinge Quadrox‐i Adult^[^ ^58^ ^]^	5700	NA	49% O_2_, 2.9 L/min	2.4% (NA)	NA	77 (NA)	3.5	Human	NA	1.7^b)^

^a)^ Values shown as “uncorrected (corrected)”

^b)^ Getinge states that the utilization period of the Quadrox‐i is limited to 6 hours. “NA” is used to denote values for which there was not sufficient data reported in the studies to calculate corrected oxygen transfer.

In three 24‐h large animal studies, we have monitored oxygen transfer, pressure drop, and hemolysis, three key performance metrics for future applications of this device in an ECMO system. The oxygen transfer rate in our device matches the computational prediction, in the range of 3.3 to 4 vol% at the 750 mL min⁻^1^ operational blood flow rate. We have achieved higher levels of oxygen transfer in the laboratory through the application of oxygen back pressure, but have not yet implemented this feature in animal studies. Pressure drop across the device is maintained throughout the 24‐h study, with some variation observed in the measured value but no perceptible trend of increasing pressure as would be expected if thrombus formation were significant enough to begin blocking flow. It is of particular significance that the PfHb remained low, suggesting minimal levels of damage to erythrocytes‐ an important clinically relevant marker of device safety. The spike in PfHb for Study 3, and the apparent rise in PfHb from 6 to 24 h in Studies 1 and 2, do not necessarily reflect a clinically meaningful elevation of free hemoglobin, in our view; interpretation of this data is limited by the free hemoglobin measurement methodology. We utilized the FDA‐approved CLIA HemoCue® Plasma/Low Hb System platform, a choice motivated by the utilization of this instrument in clinical ECMO centers, where the diagnostic threshold for clinically significant hemolysis is PfHb > 50 mg dL⁻^1^.^[^ ^55^, ^60^ ^]^ Deleterious outcomes are typically associated with free hemoglobin levels > 100 mg dL⁻^1^.^[^ ^61^ ^]^ The HemoCue® system measures PfHb using the azide methemoglobin method with corrective wavelength and turbidity compensation.^[^ ^62^ ^]^ The device is primarily rated to identify hemolysis greater than 30 mg dL⁻^1^, with caution in interpretation and differentiation of readings from 0–30 mg dL⁻^1^.^[^ ^63^ ^]^ Our objective with free hemoglobin assessment in this pilot study was identification of clinically significant hemolysis (not observed across the three studies) versus observed fluctuations in hemoglobin below this diagnostic threshold.

Systemic anticoagulation levels were high, maintaining ACT levels above those that would typically be targeted in ECMO, and therefore future studies with devices that have received an antithrombotic coating and other potential improvements will probe an expanded set of metrics related to hemocompatibility. With the very significant scaling and increased throughput reported here, our system has moved into the range required for clinical relevance for pediatric patients and for adults receiving partial lung support via extracorporeal CO_2_ removal or ECCO_2_R,^[^ ^54^, ^55^, ^64^ ^]^ although further optimization and testing will be needed.

Scale is a critically important challenge addressed by the present study, and the higher flow rates achieved here will permit future studies to further explore measures of hemocompatibility in direct comparisons with HFMO devices at equivalent blood flows. In spite of the promise of microfluidic oxygenators, prior studies with these devices have resulted in very abbreviated experiments (5 h by Gimbel et al.,^[^ ^45^ ^]^ 2 h by Thompson et al,^[^ ^44^ ^]^ and 3.5 h by Dabaghi et al.^[^ ^35^ ^]^) Further, these prior reports, some with the application of antithrombotic coatings and or nitric oxide administration even to achieve those short duration demonstration studies, an aspect that raises concerns regarding the requirement for long‐term blood stability and mitigation of thrombus formation in the microfluidic platform. Here, in the absence of any anti‐thrombotic coating on any surface of the device while utilizing systemic anticoagulation with heparin, we have demonstrated a proof‐of‐concept 24‐h operation in a very large‐scale device. This increase in scaling and blood stability is enabled by the implementation of a fully 3D microchannel network geometry with smooth, tapered transitions and branches that provides high blood flow rates while recapitulating key aspects of the in vivo microcirculation in terms of flow patterns and shear stress.

In order to guide our design of the current BLOx device, we developed a computational model that informed design decisions for optimizing blood oxygenation and carefully sculpting the hemodynamics in the transition regions and in the distribution channel network. Using data from previous studies employing earlier generation devices,^[^ ^52^, ^57^ ^]^ we developed and trained a computational model of microchannel blood oxygenation that would allow us to rationally choose an optimum set of channel dimensions consistent with oxygen transfer rate targets while subject to the shear stress and pressure drop constraints imposed on the design. The experimental results in Figure 3 validate the predictions of the model around the target flow rate of 100 mL min⁻^1^, but is somewhat less predictive at lower flow rates, particularly for the 400 mm Hg back pressure condition. This is most likely the result of the model not adequately accounting for saturation of the blood, resulting in an over‐prediction of oxygen transfer when the blood is close to saturation, as is the case at low flow rates. Future versions of the model will be modified to adequately account for this behavior.

Beyond the ability to efficiently oxygenate a patient's blood, another important feature of ECMO devices is the ability to operate consistently over extended periods of time without failure of the device or harm to the patient, both of which require creating a flow path that is not only amenable to high oxygen transfer efficiency, but also one that is designed with blood health in mind that is tolerant to perturbations, fouling, and clogging. The design presented here is the logical extension of our previous work, based on a design philosophy that posits that maintenance of uniform shear stress within a physiological range throughout the flow path improves blood health outcomes in ECMO devices even in the absence of anti‐thrombogenic coatings. In our previous design, this approach was implemented on the oxygenation channels and the on‐layer distribution network of the flow path, but did not uniformly extend to all parts of the device. In particular, the inlet and outlet regions of the layers that interface with external tubing via stainless steel hypo tubes were areas where the blood was exposed high shear stress gradients, which are known to adversely affect blood health,^[^ ^65^ ^]^ as it transitions from a circular tube to a rectangular duct. The current design takes a more holistic approach to sizing and shaping each area of the flow path that ensures the blood is exposed to a very narrow range of shear stress (7–35 dynes/cm^2) at nearly all points in the flow path with no sharp gradients^[^ ^65^ ^]^ or oscillations. While we considered platelet activation and sensitization as a driving factor in determining the ramp geometry and the total shear load of the device, the peak shear stress limit^[^ ^48^ ^]^ was set by the desire to remain in a thrombin dominant platelet aggregation regime (<35 dynes/cm^2^) rather than a von Willebrand Factor regime^[^ ^66^ ^]^ (>50‐100 dynes/cm^2^). The thrombin regime theoretically allows us to limit platelet aggregation with heparin‐based strategies, both systemic dosage and bound surface coatings. The lower limit of 7 dynes/cm^2^ theoretically allows us to reduce material induced trauma or recirculation‐like effects which exhibit as increased complement activation.^[^ ^67^ ^]^ The cross‐section of each portion of the flow path was chosen to meet the above criteria and the shape of transition regions between channels of different dimensions was achieved via an iterative process involving analytical estimates of shear stress, updating of the 3D CAD model and verification with computational fluid dynamics (CFD)in order to carefully sculpt these regions to avoid any sudden changes in shear. This approach is most clearly demonstrated in the improvements made to the layer inlet/outlet transition areas as show in Figure S8 (Supporting Information) comparing the wall shear stress in the current design to the that of our previous work. Another key aspect to this new design is the introduction of multiple banks of shorter oxygen transfer channels, a features that enables maintenance of low blood pressure drop as the device is scaled to larger flow rates. This approach is enabled by the use high‐precision micromachining, which is capable of generating molds with sufficiently complex 3D channel geometries to replicate the smooth transitions between different length scales inherent in our design, whereas soft lithography, which serves as the basis for most microfluidic ECMO devices, is primarily limited to generating structures that are 2D in nature, and 3D printing, which has not yet demonstrated the resolution, dimensional stability, or biocompatibility over the large areas and multiple length scales required to adequately replicate our design.

Increasing the pressure of the oxygen sweep gas has proven to be a simple and effective means of enhancing the oxygenation performance of the device. During the in vitro testing, this came at the cost of an unexpectedly large increase in pressure drop across the layers due to the membrane deflection caused by the differential pressure between the oxygen and blood channels, resulting in an increase in channel resistance. While we had originally expected some degree of membrane deflection, our initial attempts to model this phenomenon had suggested that this would not be a significant cause for concern. However, the model did not account for the complex interaction of the blood‐side pressure and the degree of deflection‐induced resistance at individual overlap areas throughout the length of the blood channel. Therefore, the animal studies which utilized devices built with the initial oxygen layer design were conducted without applied oxygen back pressure, while a design modification was explored for future generation devices. Using this approach, we were able to achieve oxygenation comparable with our modeling predictions and our in vitro results without applied oxygen back pressure, giving us confidence that the device was behaving as expected in vivo, and that we would be able to achieve our oxygen transfer target in future animal studies with full oxygen pressure applied. To address this issue directly, in parallel with the animal experiments, we redesigned the oxygen‐side of the mold to reduce the dimensions of the overlap area in each channel without decreasing the overall transfer area by doubling the number of oxygen channel while reducing their width by half, resulting in a greatly reduced pressure drop across the device (Figure 4, Figure S5, Supporting Information). Furthermore, we are also exploring options to strengthen the membrane with a support structure similar that reported elsewhere^[^ ^68^ ^]^ in which PDMS is cast around a thin mesh to minimize membrane deflection under pressure. The combination of these approaches will allow for even greater oxygenation efficiency at low blood pressure drop.

In three large animal studies, the BLOx device demonstrated two major results: 1) Stable and consistent performance over 24 h and 2) Meeting or exceeding the predicted/designed oxygen transfer performance. The device achieved stable 750 mL min⁻^1^ blood flow rate, stable pressure drop at ≈51 mm Hg and stable oxygen transfer near 3.5 vol% (27 mL O_2_/min). This surpasses flow rates of recently published microfluidic ECMO devices by over 9x, while maintaining clinically‐acceptable pressure drops in the blood channel for more than 7x longer than reported in the literature.^[^ ^35^ ^]^ The multi‐banked design and parallel stacking of device layers are responsible for the low observed pressure drop, while the consistent pressure drop over the 24‐h duration of the experiments suggests limited clot accumulation within the device channels despite lack of surface coating. An important consideration in evaluating the blood health associated with a new technology such as BLOx is the potential for embolization in the animal, a significant risk with current ECMO procedures. Ongoing studies are aimed at evaluation of these aspects of blood health in our system relative to HFMO devices, and will be detailed in future reports. Future studies will also invoke further scaling of the devices, and will investigate these microfluidic designs in combination with antithrombotic surface coatings in experiments where systemic heparin dosing and ACT levels are held consistent between the microfluidic oxygenators and HFMO controls.

## 4 Conclusions

Here we have demonstrated the first microfluidic respiratory assist device ever to achieve a clinical scale in terms of blood flow rate, with three microfluidic oxygenators operated in swine at a blood flow of 750 mL min⁻^1^. These large‐scale devices maintained low, stable blood pressure drop across a 24‐h duration, a milestone that also represents a first in terms of the durability and stability of a microfluidic device in an extracorporeal application (Table 1). The central innovation reported here is the use of large‐scale microfluidic layer designs comprising fully 3D branching networks of microchannels, both within the individual layers as well as the vertical blood distribution manifolds connecting the layers. These results, particularly in light of the absence of an anti‐thrombogenic coating on the device surfaces and the use of a systemic heparinization protocol, represent a promising innovation in the field of ECMO technology. An extended comparison of the physiological and coagulation effects of the BLOx devices described here in comparison with conventional HFMOs is underway and will be the subject of a future publication. Moving forward, we anticipate that further scaling of the 3D branching architecture, combined with surface functionalization treatments to the oxygenator and other circuit components, will result in continued advances toward long‐term full‐scale adult oxygenator platforms that contribute to broader use of lifesaving ECMO treatments for patients suffering from acute and chronic lung diseases.

## 5 Experimental Section

Unless otherwise noted, all reported values of oxygen transfer rate and vol% were corrected to a standardized set of blood parameters (see Supporting Information 1).^[^ ^52^ ^]^

### Device Design—Modeling

A single layer BLOx device was comprised of two molded halves: one for blood and one for oxygen, separated by a gas permeable membrane. An iterative design procedure described below was used to identify multiple channel configurations for a single‐inlet/single‐outlet blood layer capable of achieving our target performance specification of 5 vol% oxygen transfer at a flow rate of 100 mL min⁻^1^ layer. The COMSOL model was set up as described in Supporting Information 2. Using data from previous iterations of devices^[^ ^45^, ^52^, ^57^ ^]^ with varying thickness membranes and back pressures, the model was tuned to precisely match the device performance by adjusting the membrane permeability and using an apparent oxygen diffusivity and solubility in blood (as opposed to using literature values, see Supporting Information 2 for details). Because devices were tested that had different channel heights (65 µm vs 200 µm), lengths (4.7 and 8 cm), and various measured membrane thicknesses (50–100 µm), and were tested at different back pressures, the oxygen diffusivity and solubility of both the membrane and the blood could be independently back‐calculated to create a model that precisely matched the data from all testing. Note that all channel widths were previously set to 500 µm, due to manufacturing constraints; this value was never altered.

This model was then run with a series of channel lengths, heights, number of channels, membrane thicknesses, back pressures, and flow rates. The outputted oxygen transfer from each condition was then plotted to determine trends. It was discovered that total channel length (number of channels times the channel length) determined the oxygen transfer, significantly simplifying device design. Therefore the total channel length was plotted and an equation extracted for vol% oxygen transfer versus total length. For a given membrane thickness and desired vol% transfer, a polynomial curve fit was used between data points to determine the required total channel length for a given channel height and flow rate.

The target oxygen transfer and flow rate per layer were set at 5 vol% at 100 mL min⁻^1^, respectively. Because higher back pressure results in a smaller overall layer size due to the higher rate of oxygen transfer, it was decided to design the final device based on the use of 400 mmHg back pressure.

To determine the maximum length of an individual channel (and therefore determine the number of channels required as *L_total_/L_channel_*) for a given flow rate and channel height, a maximum pressure drop of 50 mmHg per channel (excluding manifolds) was set. The maximum length of a channel was then determined using the formula for pressure drop in a rectangular channel (*h* < *w*),

(1) $$\Delta P=\frac{12\eta LQ}{\left(1-0.63\frac{h}{w}\right)h^{3}w}$$

where Δ*P* is the pressure drop, *η* is the dynamic viscosity, L is the channel length, Q is the flow rate, h is the channel height and w is the channel width.^[^ ^69^ ^]^ The above expression is an approximation for a rectangular duct, and the error associated with this approximation is less than 0.2%. The shear stress was computed numerically based on a series expansion method of the velocity profile, as a function of the channel geometry and flow rate.^[^ ^70^ ^]^ The total channel length was divided by the individual channel length to obtain the number of channels needed.

As many channel height/length combinations fit this criteria, a further design constraint was added; minimize the device area. Using values for the maximum device width of 10 inches and assuming 400 µm between channels, the number of channels per width was calculated, and a number of banks calculated. This narrowed down designs to a series of 3 designs, with three, four, or five banks, all which had very similar minimum areas. Four banks were chosen as the final design due to the possibility of keeping a bifurcating manifold. Additionally, to ensure shear was in the correct range, the channel height was increased slightly to 160 µm and the total channel length recalculated to ensure adequate oxygen transfer. The final device length was calculated as follows: L_device_ = 2*(w_singlemanifold_) + (N_bank_‐1)*w_manifoldpair_ + N_bank_*L_channel_. This final design was then modeled at various flow rates and back pressures to predict device performance at other back pressures and flow rates; the final result is shown in Figure 3 compared to data.

Additional design constraints included a maximum 50 mmHg pressure drop, 3.5 Pa maximum shear stress, 50 µm thick membrane, and 10 in maximum layer width. This effectively limited the number of parallel channels that span the layer to 184, allowing room for distribution channels and entrance/exit regions. In order to maintain the hemocompatibility of our design, an additional set of rules were applied across the device to optimize the flow‐induced shear stresses imparted on the blood cells and key plasma proteins. As shear forces increase, so does the risk of activating platelets, enabling shear‐modulated platelet binding regimes, and triggering the release of pro‐thrombotic agents. Alternatively, low shear rates, caused by recirculation or low flow rates, can result in stasis‐like conditions or prolonged exposure to the device's foreign material surfaces, which promote platelet aggregation and the release of pro‐inflammatory factors. Balancing these effects can minimize the thrombogenicity of the channel design and eliminates the presence of cell damaging shear forces which occur far above the shear thresholds discussed here. Previous microfluidic oxygenators have been designed to target shear stress magnitude and exposure combinations thought to have low blood trauma risk due to conservative extrapolations from platelet shear response studies.^[^ ^38^, ^49^ ^]^ However, the available data to inform these trauma risk thresholds have significantly improved since their initial compilation, and the additional derived rules were used to constrain our design to improve hemocompatibility. Conservative thresholds were chosen for the design's total shear stress load and transitional shear ramp rate; 1000 dyne*s/cm^2^ for durations greater than 10 seconds and <170 dynes/cm^2^/s respectively.^[^ ^65^, ^71^ ^]^ A maximal shear stress target of 35 dynes/cm^2^ was set to minimize von Willebrand factor modulated platelet aggregation and maintain a thrombin dominate regime that can be addressed with a heparin‐based anticoagulation strategy^[^ ^48^, ^66^ ^]^. Finally, the targeted lower shear stress limit was 7 dynes/cm^2^ was applied to limit complement activation.^[^ ^72^ ^]^ We note that a risk incurred in the design of the microfluidic oxygenator is that optimization of the shear, pressure and flow distribution in the pristine, as‐designed network can be altered once a significant level of thrombus formation occurs.

External manifolds used to connect individual layers in the 8‐layer devices (as described below) were designed as a three‐level bifurcating manifold (1‐to‐8). Murray's law was used to define the branch diameters using the inner diameter of layer entrance/exit port as the basis for sizing the other channels and the same shear stress target range described above for the individual oxygenation layers. An iterative CAD‐CFD process was used to shape the transition from one level to the next in order to minimize low‐flow regions and shear gradients. Additional details can be found in the Supporting Information (Figure 6, Table S3, Supporting Information).

### Device Design—Mold Machining

Molds for casting the vascular and oxygen channel networks were created by milling the negative of the designed channel architecture described above into an aluminum block. A CNC machine (VU50A with FANUC 16i‐M, Misui Seiki, Franklin Lakes, NJ) using various end mills was used to remove aluminum from the block and leave behind the trunk manifold and channel structure. The blocks were then gold sputtered coated by AccuCoat Inc. (Rochester, NY)

A pair of identical molds for the manifolds were also milled out of Aluminum with the same technique described above. Tapped holes were placed in the mold at 5 different spots. This was to allow for pins to be placed in the mold in order to allow for alignment features to be cast into the manifold casted parts. The manifold molds were gold sputter coated using a KDF Sputterer (Rockleigh, NJ).

### Device Fabrication—Layer Fabrication

Each layer consisted of a vascular channel part and an oxygen channel part with a PDMS membrane sandwiched between the two. The vascular and oxygen parts were cast by pouring 550 g of Nusil MED6015 (Avantor, Carpinteria, CA) into each mold. The MED6015 was prepared by mixing the PDMS with curing agent in a 10:1 ratio per manufacturers specifications. The mixture was placed in a FlackTek DAC 600.1 FVZ (FlackTek,Landrum, SC) high speed mixer at 1000 RPM for 15 seconds followed by 2000 RPM for 1 minute and 15 seconds. The molds containing the PDMS were placed into a vacuum chamber in order to remove any trapped air bubbles. The parts were placed in a 65°C oven for a minimum of 4 hours to cure.

Manifold parts were cast using the same method described above. 120 g of MED6015 were used in each mold in order to create the two halves of the distribution manifold.

A layer was assembled by bonding the casted PDMS with the oxygen channel structure to a sheet of PDMS membrane (Silpuran, Wacker, Germany). A thin layer of room temperature vulcanizing (RTV) silicone (DOWSIL™ 3140 RTV, Dow Corning, Midland, MI) was applied to the channel side of the oxygen part using a ink briar in order to form a thin uniform layer of the DOWSIL. The oxygen part was placed onto the membrane and allowed to cure under weight at room temperature overnight. After removing from weight, a thin layer of DOWSIL was applied to the vascular part using the same procedure as used for the oxygen part. The vascular part was bonded to the opposite side of the PDMS membrane and allowed to cure under weight overnight. Stainless steel tubes were bonded into the inlet and outlet ports on both the vascular and oxygen channels using RTV(SS‐301AP Plastics Bonding Silicone Adhesive RTV, Silicone Solutions, Cuyahoga Falls, OH). The RTV was allowed to cure in a 65°C oven for 48 hours. Lengths of tygon tubing were inserted over the stainless steel inlet and outlet tubes. A thin coating of PDMS was added to the edge of the completed layer in order to seal them. The layers were tested for bonding strength by pressurizing the vascular layer to 400 mmHg for 5 min. A final leak test was also performed by flowing water through the vascular layer at 100 mL/min for 5 minutes. Leaks were checked for at the inlet and outlets and any sign of membrane tear.

Manifolds were fabricated by bonding the two halves of casted manifold parts using the DOWSIL. One part was placed on the manifold mold with alignment pins placed in the mold. A thin layer of the DOWSIL was applied to the second part using an ink briar to evenly apply the DOWSIL. The matching alignment holes in the second part were used to align the two parts together in order to create the manifold channels. The bonded manifold was cured in the oven at 65°C for a minimum of 4 hours. Stainless steel tubes were secured to the manifold inlet and outlet using the SS‐301AP RTV and allowed to cure in the oven at 65°C for 48 hours.

### Device Fabrication—Device Assembly

Each device was comprised of eight layers and two manifolds. The layers were placed into specially designed trays in sets of two. The trays interconnected to form a complete 8 layer device. The trays were designed to allow the inlet and outlet tubes to enter and exit from the tray and support the length of the layer. The blood tubes were directed and supported around a 180° turn from the layers to the manifolds. The manifolds were mounted to the sides of the completed tray stack. The oxygen tubes were connected in a “parallel” configuration in order to provide equal oxygen flow to each layer.

### Device Fabrication—Metrology

Metrology to assess dimensional accuracy was conducted on the master molds for the vascular and oxygen layers, and on the casted replicated silicone layers. The latter measurements were conducted both profilometers and SEM characterization to ensure that the dimensions of the silicone channels (depth and width) did not vary significantly from the mold geometry. The SEM was also used to assess the tapered and ramped regions of the distribution manifolds to confirm that the designed transition regions were accurately replicated during the machining process.

### In Vitro Blood Testing—Blood Oxygen Transfer Testing

Donor swine blood (Lampire) was warmed to 37C and corrected to 65% SO2 +/‐ 3% before being perfused through single‐layer BLOx devices to evaluate oxygen transfer. Blood flow rates ranged between 25 mL/min and 150 mL/min and were controlled with a peristaltic pump (Watson Marlow). 100% oxygen gas was delivered to the oxygen microchannels and controlled with a mass flow controller (Alicat Scientific). Oxygenation status of the blood was measured using a blood‐gas analyzer (BGA, Instrumentation L) and a co‐oximetry assessment using an Avoximeter (Werfen). Blood was sampled pre‐ and post‐device for accurate assessment of blood oxygenation at each flow rate and applied back pressure tested.

Oxygen back pressure was applied by setting a dual‐valve pressure controller (Alicat) to the oxygen outlet of the oxygenator test circuit. 100 mL/min oxygen continued to flow through the oxygen channels while a back pressure of 200 mm Hg or 400 mm Hg was controlled while sweeping blood flow rates.

Pressure in both the vascular and oxygen microchannels was measured in real time with BIOPAC pressure transducers and data acquisition systems. Transducers were placed at the vascular and oxygen entrance ports. Sampling rate was set to 50 Hz.

### In Vitro Blood Testing—Pressure Drop Measurement

Pressure drop across the microfluidic oxygenator was monitored using a pressure sensor (Pendotech, Princeton NJ) monitoring the inlet pressure at the vascular channel entry point. Pressure drop in mm Hg was monitored as a function of the blood flow rate set for the peristaltic pump. The measured pressure drop included the microfluidic vascular network and the inlet and outlet tubing connections.

### Experimental Set‐Up—Animal Preparation

The animal study was carried out at the AREVA laboratory, in compliance with the Animal Welfare Act, the principles of the *Guide for the Care and Use of Laboratory Animals*, and all local, state, and federal guidelines for ethical use of animals. In conducting research using animals, the investigators adhere to the laws of the United States and regulations of the Department of Agriculture. The University of Texas Health Science Center San Antonio (San Antonio, TX) Institutional Animal Care and Use Committee approved all research conduct (Protocol SU0050824). Secondary level animal use protocol approvals were obtained from the Department of the Army Animal Care and Use Review Office (PR181115). Female Yorkshire swine (55 ± 5 kg) were anesthetized, intubated and volume‐control ventilated. Arterial and venous lines were placed for monitoring, administration of intravenous anesthesia medication and continuous heparin infusion. Animals received surgical plane anesthesia and analgesia for the entirety of the study.^[^ ^54^ ^]^

### Experimental Set‐Up—Extracorporeal Life Support

The extracorporeal circuit incorporating the BLOx microfluidic oxygenator was constructed using 1.3 m length tubing connections (0.635 cm ID) at the pre‐membrane inlet and post‐membrane outlet. A centrifugal pump (DP3 pump head with Novalung Console; Fresenius Medical Care; Bad Homburg, Germany) was integrated into the pre‐membrane tubing line. A clamp‐on flow sensor (ME6PXL; Transonic Systems, Ithaca, NY) was placed on the post‐oxygenator tubing line for monitoring blood flow rate. Pressure sensors compatible with the Novalung Console (Fresenius Medical Care; Bad Homburg, Germany) were placed at the pre‐ and post‐membrane tubing ports for monitoring of circuit pressures. The entire extracorporeal circuit including the BLOx membrane was primed with heparinized saline (5000 U /L) at a flow rate of 750 mL min⁻^1^ for a minimum duration of 1 h to remove air from the system prior to start of cannulation. A photograph of the device during priming is illustrated in Figure S9a (Supporting Information).

Prior to start of cannulation, a 4000 U unfractionated heparin (UFH) bolus was administered. A veno‐venous jugular dual lumen catheter was placed for initiation of veno‐venous ECLS. The catheter was placed percutaneously into the right jugular vein and the connected to the microfluidic oxygenator in an air‐free fashion. Blood flow was then initiated through the BLOx device at a flow rate of 750 mL min⁻^1^. Sweep gas (100% oxygen) was initiated at a flow rate of 4 L min⁻^1^ (the first animal study started at 1 L min⁻^1^ sweep gas and was increased to 3 L min⁻^1^ during the last hour, while the other two studies were conducted at 4 L min⁻^1^ sweep throughout). Following cannulation and start of extracorporeal circulation, UFH was infused continuously at a starting rate of 40 U kg⁻^1^ h⁻^1^ and titrated to achieve an ACT of 250–350 s.

Blood samples were collected to determine oxygen transfer and plasma‐free hemoglobin (Hemocue AB; Angelhom, Sweden). Circuit pressures and flow rates were recorded hourly. A photograph of the device during Animal Study #1 is depicted in Figure S9b (Supporting Information).

## Conflict of Interest

The authors declare no conflict of interest.

## Supporting information

Supporting Information

## Data Availability

The data that support the findings of this study are available from the corresponding author upon reasonable request.

## References

[1] WHO Global Health Estimate Report – WHO Newsroom.

[2] Z. J. Lim , A. Subramaniam , M. Ponnapa Reddy , G. Blecher , U. Kadam , A. Afroz , B. Billah , S. Ashwin , M. Kubicki , F. Bilotta , J. R. Curtis , F. Rubulotta , Am J. Respir. Crit. Care Med. 2021, 203, 54. 33119402 10.1164/rccm.202006-2405OC PMC7781141

[3] E. Fan , D. Brodie , A. S. Slutsky , Am J. Respir. Crit. Care Med. 2017, 195, 1137. 28459339 10.1164/rccm.201702-0292ED

[4] W. Federspiel , K. Henchir , in Encyclopedia of Biomaterials and Biomedical Engineering, 2nd ed., Informa Healthcare, New York 2008.

[5] E. C. Goligher , N. D. Ferguson , L. J. Brochard , Lancet 2016, 387, 1856. 27203509 10.1016/S0140-6736(16)30176-3

[6] A. Combes , D. Hajage , G. Capellier , A. Demoule , S. Lavoué , C. Guervilly , D. Da Silva , L. Zafrani , P. Tirot , B. Veber , E. Maury , B. Levy , Y. Cohen , C. Richard , P. Kalfon , L. Bouadma , H. Mehdaoui , G. Beduneau , G. Lebreton , L. Brochard , N. D. Ferguson , E. Fan , A. S. Slutsky , D. Brodie , A. Mercat , N. Engl. J. Med. 2018, 378, 1965. 29791822 10.1056/NEJMoa1800385

[7] R. H. Bartlett , ASAIO J. 2017, 378, 832. 10.1097/MAT.0000000000000697 29084039

[8] P. P O'rourke , R. K. Crone , J. P. Vacanti , J. H. Ware , C. W. Lillehei , R. B. Parad , M. F. Epstein , Pediatrics 1989, 84, 957. 2685740

[9] P. P. O'Rourke , C. J. Stolar , J. B. Zwischenberger , S. M. Snedecor , R. H. Bartlett , J. Pediatr. Surg. 1993, 28, 523. 8483064 10.1016/0022-3468(93)90610-w

[10] J. M. Toomasian , S. M. Snedecor , R. G. Cornell , R. E. Cilley , R. H. Bartlett , ASAIO Trans. 1988, 34, 140. 3370175 10.1097/00002480-198804000-00011

[11] B. W. Gray , J. W. Haft , J. C. Hirsch , G. M. Annich , R. B. Hirschl , R. H. Bartlett , ASAIO J. 2015, 34, 2. 10.1097/MAT.0000000000000150 PMC4280306 25251585

[12] G. J. Peek , M. Mugford , R. Tiruvoipati , A. Wilson , E. Allen , M. M. Thalanany , C. L. Hibbert , A. Truesdale , F. Clemens , N. Cooper , R. K. Firmin , D. Elbourne , Lancet 2009, 374, 1351.19762075

[13] M. A. Noah , G. J. Peek , S. J. Finney , M. J. Griffiths , D. A. Harrison , R. Grieve , M. Z. Sadique , J. S. Sekhon , D. F. Mcauley , R. K. Firmin , C. Harvey , J. J. Cordingley , S. Price , A. Vuylsteke , D. P. Jenkins , D. W. Noble , R. Bloomfield , T. S. Walsh , G. D. Perkins , D. Menon , B. L. Taylor , K. M. Rowan , JAMA 2011, 306, 1659. 21976615 10.1001/jama.2011.1471

[14] R. P. Barbaro , G. Maclaren , P. S. Boonstra , A. Combes , C. Agerstrand , G. Annich , R. Diaz , E. Fan , K. Hryniewicz , R. Lorusso , M. L. Paden , C. M. Stead , J. Swol , T. J. Iwashyna , A. S. Slutsky , D. Brodie , Lancet 2021, 398, 1230. 34599878 10.1016/S0140-6736(21)01960-7 PMC8480964

[15] R. P. Barbaro , G. Maclaren , P. S. Boonstra , T. J. Iwashyna , A. S. Slutsky , E. Fan , R. H. Bartlett , J. E. Tonna , R. Hyslop , J. J. Fanning , P. T. Rycus , S. J. Hyer , M. M. Anders , C. L. Agerstrand , K. Hryniewicz , R. Diaz , R. Lorusso , A. Combes , D. Brodie , P. Alexander , N. Barrett , J. Bělohlávek , D. Fisher , J. Fraser , A. A. Hssain , J. S. Jung , M. Mcmullan , Y. Mehta , M. T. Ogino , M. L. Paden , et al., Lancet 2020, 396, 1071.32987008

[16] M. Schoberer , J. Arens , A. Lohr , M. Seehase , R. K. Jellema , J. J. Collins , B. W. Kramer , T. Schmitz‐Rode , U. Steinseifer , T. Orlikowsky , Artif. Organs 2012, 36, 512. 22309513 10.1111/j.1525-1594.2011.01404.x

[17] D. Palanzo , F. Qiu , L. Baer , J. B Clark , J. L. Myers , A. Ündar , Artif. Organs. 2010, 34, 869. 21092028 10.1111/j.1525-1594.2010.01127.x PMC3066638

[18] D. Brodie , A. S. Slutsky , A. Combes , JAMA 2019, 322, 557. 31408142 10.1001/jama.2019.9302

[19] A. J. Doyle , B. J. Hunt , Front. Med. 2018, 5, 352. 10.3389/fmed.2018.00352 PMC6299009 30619862

[20] J. Sniderman , P. Monagle , G. M. Annich , G. Maclaren , Res. Pract. Thromb. Haemost. 2020, 4, 455. 32548547 10.1002/rth2.12346 PMC7292669

[21] B. Nagler , A. Hermann , O. Robak , P. Schellongowski , N. Buchtele , A. Bojic , M. Schmid , Z. Zauner , M. P. Winter , G. Heinz , R. Ullrich , F. Kraft , D. Wiedemann , M. H. Bernardi , T. Staudinger , W. Lamm , ASAIO J. 2021, 67, 776. 34170882 10.1097/MAT.0000000000001332

[22] S. B. Patel , J. P. Kress , Am J. Respir. Crit. Care Med. 2012, 185, 486. 22016443 10.1164/rccm.201102-0273CI

[23] K. Adrian , K. Mellgren , M. Skogby , L. G. Friberg , G. Mellgren , H. Wadenvik , Artif. Organs 1998, 22, 859. 9790084 10.1046/j.1525-1594.1998.06121.x

[24] J. Graulich , J. Sonntag , M. Marcinkowski , K. Bauer , H. Kössel , C. Bührer , M. Obladen , H. T. Versmold , Mediators Inflammation 2002, 11, 69. 10.1080/09629350220131908 PMC1781648 12061426

[25] J. Graulich , B. Walzog , M. Marcinkowski , K. Bauer , H. Kössel , G. Fuhrmann , C. Bührer , P. Gaehtgens , H. T. Versmold , Pediatr. Res. 2000, 48, 679. 11044491 10.1203/00006450-200011000-00021

[26] J. E. Millar , J. P. Fanning , C. I. McDonald , D. F. McAuley , J. F. Fraser , Crit. Care 2016, 20, 387. 27890016 10.1186/s13054-016-1570-4 PMC5125043

[27] T. R. Roberts , G. T. Harea , P. Singha , K. N. Sieck , B. M. Beely , D. S. Wendorff , J. H. Choi , S. Ande , H. Handa , A. I. Batchinsky , ASAIO J. 2020, 66, 809. 31453831 10.1097/MAT.0000000000001055

[28] T. R. Roberts , M. R. S. Garren , H. Handa , A. I. Batchinsky , J. Trauma Acute Care Surg. 2020, 89, S59. 32251267 10.1097/TA.0000000000002700 PMC7398848

[29] T. R. Roberts , R. P. Seekell , Y. Zang , G. Harea , Z. Zhang , A. I. Batchinsky , Perfusion 2022, 10.1177/02676591221095469. 35514052

[30] H. Appelt , A. Philipp , T. Mueller , M. Foltan , M. Lubnow , D. Lunz , F. Zeman , K. Lehle , PLoS One 2020, 15, e0227793. 31986168 10.1371/journal.pone.0227793 PMC6984694

[31] K. Lehle , A. Philipp , T. Müller , F. Schettler , T. Bein , C. Schmid , M. Lubnow , Artif. Organs 2014, 38, 391. 24117454 10.1111/aor.12180

[32] C. Dornia , A. Philipp , S. Bauer , P. Hoffstetter , K. Lehle , C. Schmid , M. Lubnow , C. Stroszczynski , A. G. Schreyer , ASAIO J. 2013, 59, 439. 23820285 10.1097/MAT.0b013e3182976eff

[33] A. R. Mazaheri , G. Ahmadi , Artif. Organs 2006, 30, 10. 16409392 10.1111/j.1525-1594.2006.00150.x

[34] W. Sun , S. Wang , Z. Chen , J. Zhang , T. Li , K. Arias , B. P. Griffith , Z. J. Wu , Artif. Organs 2020, 44, 717. 31970795 10.1111/aor.13646 PMC7308201

[35] M. Dabaghi , N. Rochow , N. Saraei , G. Fusch , S. Monkman , K. Da , A. Shahin‐Shamsabadi , J. L. Brash , D. Predescu , K. Delaney , C. Fusch , P. R. Selvaganapathy , Adv. Sci. 2020, 7, 2001860. 10.1002/advs.202001860 PMC7610273 33173732

[36] K. A. Burgess , H.‐H. Hu , W. R. Wagner , W. J. Federspiel , Biomed. Microdevices 2009, 11, 117. 18696229 10.1007/s10544-008-9215-2

[37] J. A. Potkay , M. Magnetta , A. Vinson , B. Cmolik , Lab Chip 2011, 11, 2901. 21755093 10.1039/c1lc20020h

[38] J. A. Potkay , Lab Chip 2014, 14, 4122. 25198427 10.1039/c4lc00828f

[39] T. Kniazeva , A. A. Epshteyn , J. C. Hsiao , E. S. Kim , V. B. Kolachalama , J. L. Charest , J. T. Borenstein , Lab Chip 2012, 12, 1686. 22418858 10.1039/c2lc21156d PMC3320667

[40] D. M. Hoganson , H. I. Pryor Ii , E. K. Bassett , I. D. Spool , J. P. Vacanti , Lab Chip 2011, 11, 700. 21152606 10.1039/c0lc00158a

[41] J. K. Lee , H. H. Kung , L. F. Mockros , ASAIO J. 2008, 54, 372. 18645354 10.1097/MAT.0b013e31817ed9e1

[42] W‐I. Wu , N. Rochow , E. Chan , G. Fusch , A. Manan , D. Nagpal , P. R. Selvaganapathy , C. Fusch , Lab Chip 2013, 13, 2641. 23702615 10.1039/c3lc41417e

[43] A. A. Gimbel , J. C. Hsiao , E. S. Kim , D. J. Lewis , T. F. Risoleo , J. N. Urban , J. T. Borenstein , Artif. Organs 2021, 45, E247. 33561881 10.1111/aor.13935

[44] A. J. Thompson , L. J. Ma , T. Major , M. Jeakle , O. Lautner‐Csorba , M. J. Goudie , H. Handa , A. Rojas‐Peña , J. A. Potkay , Acta Biomater. 2020, 112, 190. 32434076 10.1016/j.actbio.2020.05.008 PMC10168296

[45] J. A. Santos , A. A. Gimbel , A. Peppas , J. G. Truslow , D. A. Lang , S. Sukavaneshvar , D. Solt , T. J. Mulhern , A. Markoski , E. S. Kim , J. C.‐M. Hsiao , D. J. Lewis , D. I. Harjes , C. Dibiasio , J. L. Charest , J. T. Borenstein , Lab Chip 2021, 21, 4637. 34730597 10.1039/d1lc00287b

[46] G. Wagner , A. Kaesler , U. Steinseifer , T. Schmitz‐Rode , J. Arens , Lab Chip. 2016, 16, 1272. 26956695 10.1039/c5lc01508a

[47] S. P. Jackson , Blood 2007, 109, 5087. 17311994 10.1182/blood-2006-12-027698

[48] A. Rana , E. Westein , B. Niego , C. E. Hagemeyer , Front. Cardiovasc. Med. 2019, 6, 141. 31620451 10.3389/fcvm.2019.00141 PMC6763557

[49] S. N. Vaslef , R. W. Anderson , The Artificial Lung, Landes Bioscience, Georgetown, Austin 2002.

[50] C. D. Murray , Proc. Natl. Acad. Sci. U. S. A. 1926, 12, 207.16576980

[51] D. R. Emerson , K. Cieślicki , X. Gu , R. W. Barber , Lab Chip 2006, 6, 447. 16511629 10.1039/b516975e

[52] E. M. Vedula , B. C. Isenberg , J. Santos , W. Lai , D. J. Lewis , D. Sutherland , T. R. Roberts , G. T. Harea , C. Wells , B. Teece , J. Urban , T. Risoleo , D. Solt , S. Leazer , K. Chung , S. Sukavaneshvar , A. I. Batchinsky , J. T. Borenstein , ASAIO J. 2022, 68, 1312. 36194101 10.1097/MAT.0000000000001647 PMC9521578

[53] L. Lequier , S. B. Horton , D. M. McMullan , R. H. Bartlett , Pediatr. Crit. Care Med. 2013, 14, S7. 23735989 10.1097/PCC.0b013e318292dd10 PMC3742331

[54] A. I. Batchinsky , B. S. Jordan , D. Regn , C. Necsoiu , W. J. Federspiel , M. J. Morris , L. C. Cancio , Crit. Care Med. 2011, 39, 1382. 21317644 10.1097/CCM.0b013e31820eda45

[55] Extracorporeal Life Support: The ELSO Red Book 6th Edition, https://books.google.com/books?id=iX08zwEACAAJ (accessed: September 2022).

[56] T. L. Astor , J. T. Borenstein , Artif. Organs 2022, 46, 1227. 35514275 10.1111/aor.14266

[57] J. Santos , E. M. Vedula , W. Lai , B. C. Isenberg , D. J. Lewis , D. Lang , D. Sutherland , T. R. Roberts , G. T. Harea , C. Wells , B. Teece , P. Karandikar , J. Urban , T. Risoleo , A. Gimbel , D. Solt , S. Leazer , K. K. Chung , S. Sukavaneshvar , A. I. Batchinsky , J. T. Borenstein , Micromachines 2021, 12, 888. 34442512 10.3390/mi12080888 PMC8398684

[58] R. H. J. Hendrix , Y. M. Ganushchak , P. W. Weerwind , Artif. Organs 2018, 42, 611. 29473675 10.1111/aor.13084

[59] R. M. Ginther Jr. , R. Gorney , R. Cruz , Perfusion 2013, 28, 194. 23449822 10.1177/0267659113475694

[60] H. R. Omar , M. Mirsaeidi , S. Socias , C. Sprenker , C. Caldeira , E. M. Camporesi , D. Mangar , PLoS One 2015, 10, e0124034. 25902047 10.1371/journal.pone.0124034 PMC4406730

[61] N. Dufour , A. Radjou , M. Thuong , ASAIO J. 2020, 66, 239. 30985331 10.1097/MAT.0000000000000974

[62] US‐Manual_Plasma Low Hb.pdf, https://www.hemocue.us/wp‐content/uploads/2020/08/US‐Manual_Plasma_Low_Hb.pdf (accessed: November 2022).

[63] E. C. Calvaresi , S. L. La'ulu , T. M. Snow , T. R. Allison , J. R. Genzen , Int. J. Lab. Hematol. 2021, 43, 1145. 33449436 10.1111/ijlh.13457

[64] R. G Jeffries , Y. Mussin , D. S. Bulanin , L. W. Lund , E. Kocyildirim , Z. Z. Zhumadilov , F. S. Olzhayev , W. J. Federspiel , P. D. Wearden , Int. J. Artif. Organs 2014, 37, 10.5301/ijao.5000372. PMC5410869 25588763

[65] J. Sheriff , J. S. Soares , M. Xenos , J. Jesty , D. Bluestein , Ann. Biomed. Eng. 2013, 41, 1279. 23400312 10.1007/s10439-013-0758-x PMC3640664

[66] S. Gogia , S. Neelamegham , Biorheology 2015, 52, 319. 26600266 10.3233/BIR-15061 PMC4927820

[67] F. Consolo , J. Sheriff , S. Gorla , N. Magri , D. Bluestein , F. Pappalardo , M. J. Slepian , G. B. Fiore , A. Redaelli , Sci. Rep. 2017, 7, 4994. 28694489 10.1038/s41598-017-05130-5 PMC5503983

[68] H. Matharoo , M. Dabaghi , N. Rochow , G. Fusch , N. Saraei , M. Tauhiduzzaman , S. Veldhuis , J. Brash , C. Fusch , P. R Selvaganapathy , Biomicrofluidics 2018, 12, 014107. 29375728 10.1063/1.5014028 PMC5764751

[69] H. Bruus , in Microscale Acoustofluidics (Eds: T. Laurell , A. Lenshof ), The Royal Society of Chemistry, Cambridge 2014.

[70] Incompressible Flow , https://books.google.com/books?id=sa4eAAAAQBAJ (accessed: December 2022).

[71] J. Sheriff , D. Bluestein , G. Girdhar , J. Jesty , Ann. Biomed. Eng. 2010, 38, 1442. 20135353 10.1007/s10439-010-9936-2 PMC2872093

[72] S. K. Shanmugavelayudam , D. A. Rubenstein , W. Yin , Platelets 2011, 22, 602. 21679034 10.3109/09537104.2011.585257

[73] E. M. Vedula , B. C. Isenberg , J. Santos , W. Lai , D. J. Lewis , D. Sutherland , T. R. Roberts , G. T. Harea , C. Wells , B. Teece , J. Urban , T. Risoleo , D. Solt , S. Leazer , K. Chung , S. Sukavaneshvar , A. I. Batchinsky , J. T. Borenstein , ASAIO J. 2022, 68, 1312. 36194101 10.1097/MAT.0000000000001647 PMC9521578

[74] S. S. Shibeshi , W. E. Collins , Appl. Rheol. 2005, 15, 398. 16932804 10.1901/jaba.2005.15-398 PMC1552100

