# Supplementary material for: A Clinical‐Scale Microfluidic Respiratory Assist Device with 3D Branching Vascular Networks
Source: Adv Sci (Weinh). 2023 Apr 24;10(18):2207455. doi: 10.1002/advs.202207455 (PMC10288269; doi:10.1002/advs.202207455)
Supplement: Supplementary file 1 — Supporting Information [file ADVS-10-2207455-s001.pdf]

1. Supporting Information

1.1 Vol% correction

Blood parameters such as starting oxygen saturation and the hemoglobin concentration have a strong influence on the amount of oxygen that is transfered to the blood by a given device. In order to compare oxygenation performance across difference devices using different blood, it is necessary to correct oxygen transfer rate to a standardized set of starting conditions. We define this set of standard conditions as follows:

- Inlet oxygen saturation ($SO2in^*$): 65%
- Total hemoglobin concentration (tHb^*): 12g/dL
- Effective saturation limit of blood (SO2sat): 98.4%

Using these parameters, we correct the measured transfer rate and vol% to the standard conditions using the follow equations:

$$corrected\ transfer\ rate = measured\ transfer\ rate \times \frac{tHb^*}{tHb}\left(\frac{SO2sat - SO2in^*}{SO2sat - SO2_{in}}\right)$$

$$corrected\ vol\% = \frac{corrected\ transfer\ rate}{blood\ flow\ rate}$$

1.2 COMSOL model description

A COMSOL model was initially created to calculate oxygen transfer through the system using data and dimensions of a previous iteration of the device [73]–[75] that could then be used to design future iterations. The model uses 200 µm tall oxygen channels with 1mm center-to-center spacing. The rib width (between oxygen channels) is 440 µm, calculated from a nominal 500 µm width and subtracting the amount lost from the draft angle of the mold (10°). The blood channel width is fixed at 500 µm, and the membrane thickness for these calculations was fixed at 65 µm (original spun-coated Nusil membrane had a ~65 µm thickness). Commercially available Silpuran membranes at 50µm thickness were determined to have the same oxygen permeability as the 65 µm Nusil membranes, so calculations for both systems are equivalent).

The model sets the concentration of the oxygen on the PDMS surfaces in the oxygen channels by the gas pressurein the oxygen channels (760 mmHg + back pressure) multiplied by the solubility of the membrane (S_{mem}). The partition coefficient between the membrane and blood (K_{mb}) is calculated by dividing the solubility of the blood by the solubility of the membrane. This value is used to calculate the oxygen transfer between the membrane and the blood (the discontinuity in solubility at that interface equates to a discontinuity in the concentration because the partial pressure must be equal).

In total, there are four parameters that the model uses to calculate oxygen transfer; the diffusivity and solubility of the membrane and the blood. Values of diffusivity (D_{mem}, D_{eff}) and solubility (S_{mem} and S_{eff}) of the membrane and blood were previously determined empirically by fitting the modeling results to data from prior experiments that varied the channel height, length, oxygen pressure and flow rate. There is a 3 μm cell-free plasma layer that is included in the model; this layer is so thin compared to the rest of the channel that its properties can change by multiple orders of magnitude without affecting the results. The diffusivity of this layer was set to literature values for plasma in this study (see Table S1).

The flow was modeled as a laminar flow entering the channel fully developed. There is a 3 μm cell-free plasma layer that has Newtonian properties and viscosity set to 0.0035 Pa*s and density of 1025 kg/m^3.[76] The rest of the blood channel is modeled as a Carreau fluid with properties defined as shown in Table S1.

The width of the channel is fixed at 500 μm (250 um for half-width in the simulation due to symmetry plane) and the distance between ribs is similarly fixed at 1000 μm due to machining constraints. The height and length of the channel were varied to determine the optimum dimensions for oxygen transfer (given the membrane thickness, oxygen pressure, and desired blood flow). Various blood flows from 5 to 150 mL/min were run to determine the dependency of oxygen transfer on blood flow rate.

Name	Expression	Value	Description
**H_o2**	200 [um]	$2x10^{-4}$ m	oxygen channel height
**H_chan**	160[um]	$1.6x10^{-4}$ m	channel height
**W_chan**	250 [um]	$1.5x10^{-4}$ m	half width of channel (uses symmetry plane in model)
**t_mem**	65[um]	6.5 $x10^{-5}$ m	membrane thickness
**L_channel**	4[cm]/N	0.004 m	channel length (divided by N for computational efficiency)
**rib_w**	440 [um]	4.4 $x10^{-4}$ m	rib width
**rib_spacing**	1000 [um]	0.001 m	rib spacing
**array_size**	floor(l_channel/(rib_spacing))	4	number of ribs
**rbc_free**	3 [um]	3 $x10^{-6}$ m	Thickness of RBC-free layer
**Nchan**	736*N	7360	Number of channels (multiplied by N for computational efficiency)
**Qblood**	QbloodTot/(Nchan*2)	1.019 $x10^{-11}$ m^3/s	flow rate per channel (halved for symmetry plane)
**QbloodTot**	9 [cm^3/min]	1.5 $x10^{-7}$ m^3/s	Blood Flow rate
**Dmem**	1e-5 [cm^2/s]	1 $x10^{-9}$ m^2/s	membrane diffusivity 3.4e-5 [cm^2/s]
**Dplasma**	1.8e-5 [cm^2/s]	1.8 $x10^{-9}$ m^2/s	plasma diffusivity, 1.8e-5 from Potkay ref
**Deff**	4e-6 [cm^2/s]	4 $x10^{-10}$ m^2/s	Effective blood diffusivity
**Smem**	2e-5 [M/mmHg]	1.5001 $x10^{-4}$ $s^2 \cdot mol/(kg \cdot m^2)$	O2 solubility in PDMS, set to ref value of 0.31 mL/g = 2e-5M/mmHg
**Splasma**	1.339e-6 [M/mmHg]	1.0043 $x10^{-5}$ $s^2 \cdot mol/(kg \cdot m^2)$	plasma solubility, ref value 1.339e-6 [M/mmHg]
**Seff**	2.3e-5 [M/mmHg]	1.7251 $x10^{-4}$ $s^2 \cdot mol/(kg \cdot m^2)$	effective blood solubility
**Kmb**	Seff/Smem	1.15	Partition Coefficient
**Pg_gas**	200 [mmHg]	26664 Pa	gauge pressure on oxygen upstream
**P_gas**	760 [mmHg] + Pg_gas	1.2799 $x10^{5}$ Pa	oxygen pressure for conc measurement
**N**	10	10	
**Mu_plasma**	0.0035 [Pa*s]	0.0035 Pa·s	Plasma viscosity
**Mu_0**	0.056 [Pa*s]	0.056 Pa·s	Zero shear rate viscosity
**Mu_inf**	.0035 [Pa*s]	0.0035 Pa·s	Infinite shear rate viscosity
**Lamda**	3.313 [s]	3.313 s	Carreau model parameter
**nu**	0.3568	0.3568	Carreau model parameter

Table S1: *List of parameters used in the COMOSOL model.*

The simulations were run with a structured mesh in the blood layer, a free tetrahedral mesh in the membrane, and boundary layers were added in the blood and plasma layers (Figure S1). A total of 61,697 elements were used to accurately model both blood flow and oxygen transfer.

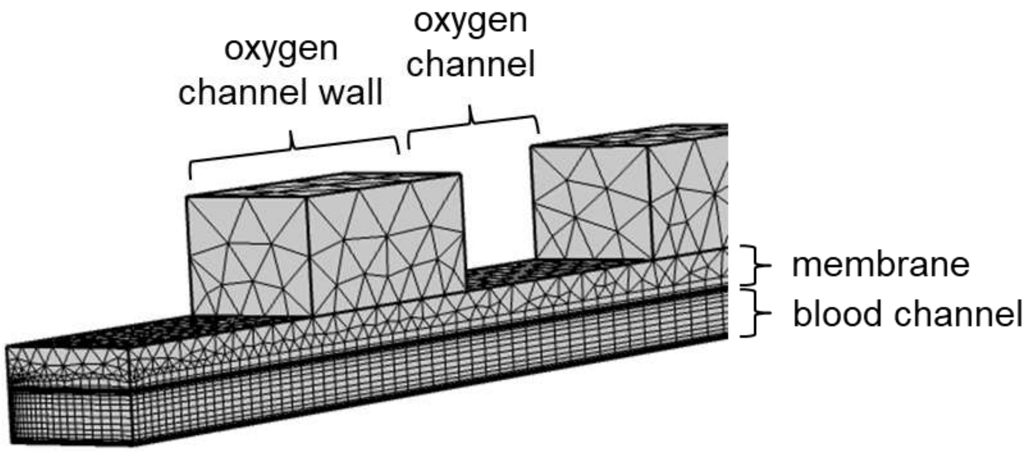

***Figure S1**: Image of the mesh distribution in the COMSOL oxygen transfer model.*

All results from the simulation were used to derive an optimum channel height and length given design constraints (discussed in methods section of main text). The number of channels per bank was fixed at 184 due to the channel width and a physical limit to the maximum overall width of the device, so the number of banks was calculated based on the required channel length. The 4- and 5-bank designs had similar overall device length (Table S2); the 4-bank was chosen to maintain a bifurcating manifold leading into the banks.

The shear rates were calculated for the 4-bank design and determined that the channel height needed to be slightly taller to reduce the shear rate in the channels. The channel height was increased from 147 μm to 160 μm, and the channel length recalculated to be 9.1 cm to accommodate the increased height.

Number of Banks	Channels per bank	Height (μm)	Channel Length (mm)	Total Device Length (mm)
**1**	184	441	60	62.7
**2**	368	254	22.9	50.7
**3**	552	184	13.1	46.6
**4**	736	147	8.9	45.2
**5**	920	123	6.7	45.1

***Table S2:** Required channel dimensions based on number of banks.*

1.3 Flowlo distribution

The flow distribution and pressure were checked with CFD. One bank of the device was modeled independently to check that the flow rate in each channel was uniform, and the manifold that distributed flow to the banks was modeled separately with a single resistive channel between the inlet and outlet manifolds for computational efficiency. Both models show uniform flow (Figures S2 and S3) and pressure drop (Figures S4 and S5) across the channels and device.

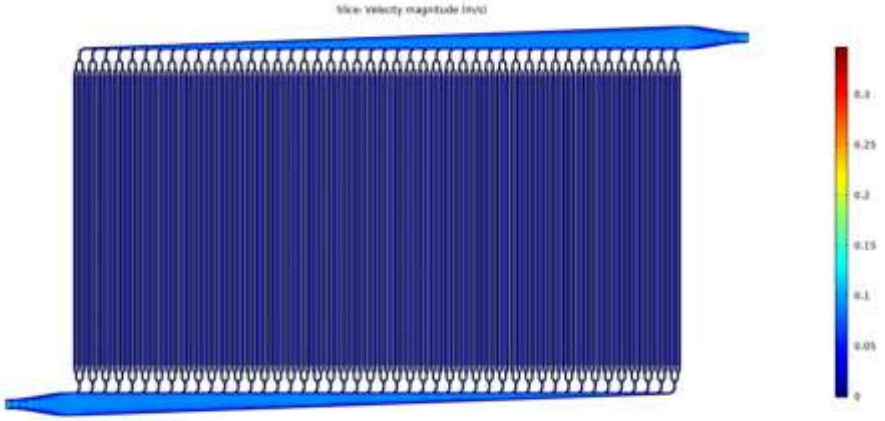

Figure S2: *COMSOL simulation showing uniform fluid velocity in a single bank of the BLOx device.*

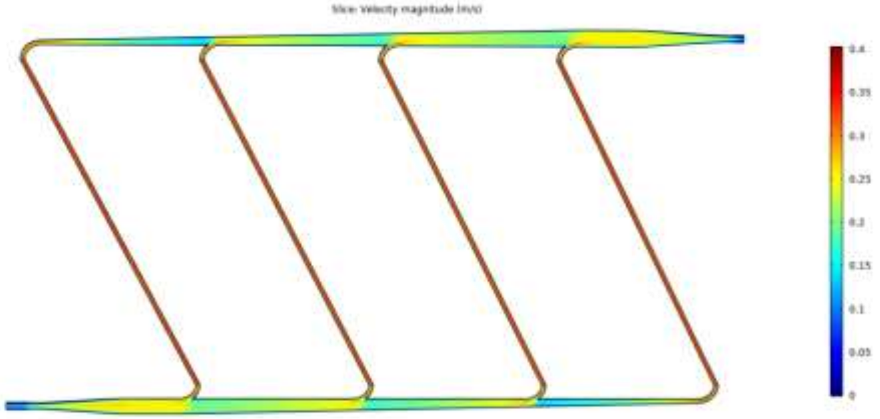

Figure S3: *COMSOL simulation showing uniform distribution to each bank of the BLOx device, using lump resistance channels in place of banks for computational efficiency.*

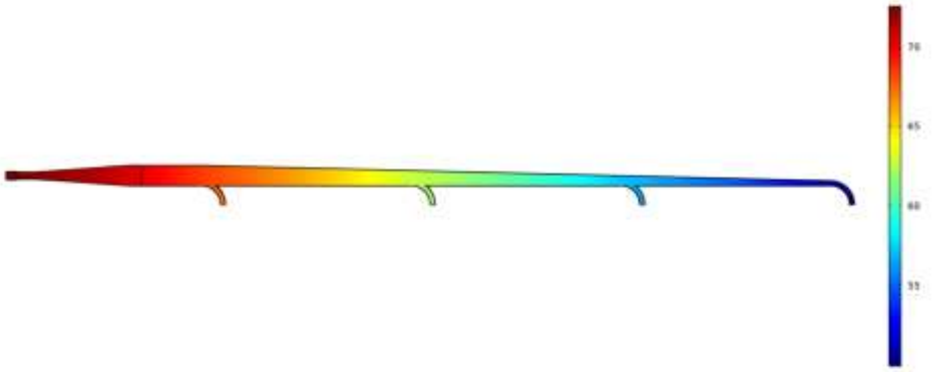

Figure S4: *COMSOL simulation showing uniform pressure gradient in the main trunk manifold of the BLOx device.*

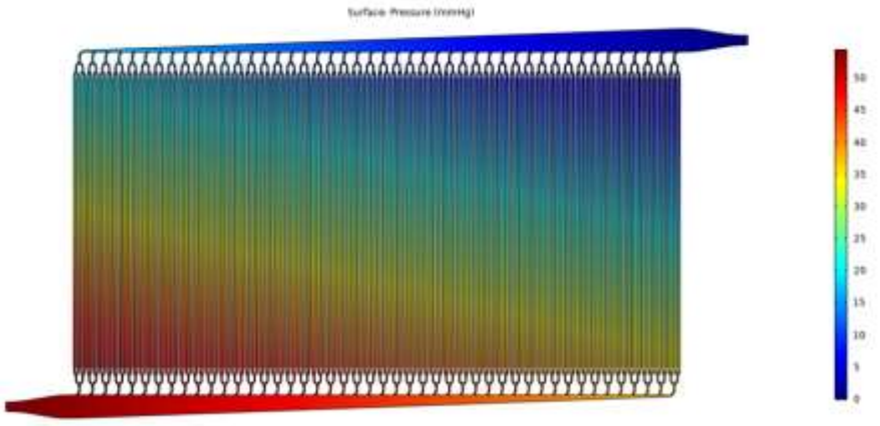

Figure S5: *COMSOL simulation showing uniform pressure drop across all channels in a single bank of the BLOx device.*

1.4 8-layer manifold dimensions and shear stress distribution

**Level**	**Diameter (mil)**
Inlet	236
Branch 1	187
Branch 2	149
Branch 3	118

Table S3: *Branching channel diameters in the 8-layer external manifold*

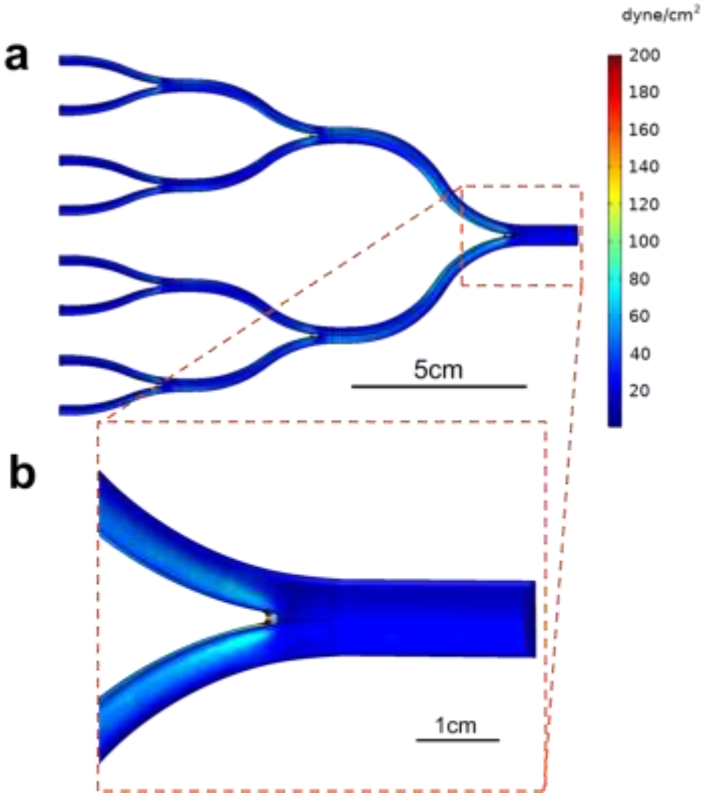

Figure S6: *Shear stress distribution in 8-layer manifold from COMSOL computational fluid dynamics model: a) full manifold and b) exploded view of a one of the bifurcations.*

1.5 Updated oxygen layer with additional membrane support features

In the original BLOx design, there was significant overlap of the oxygen channels with the wide blood distribution channels (Figure S7a), which lead to a high degree of channel deformation due to the deflection of the membranes in these regions at elevated oxygen pressures. An updated oxygen channel layer and the addition of extra membrane support structures (Figure S7b/c/d) had the effect of minimizing the membrane deflection to an acceptable level at 400mmHg, resulting in a greatly reduced pressure drop across the device (original layout = 367 mmHg, updated layout = 110 mmHg) at the nominal flow rate of 100 mL/min.

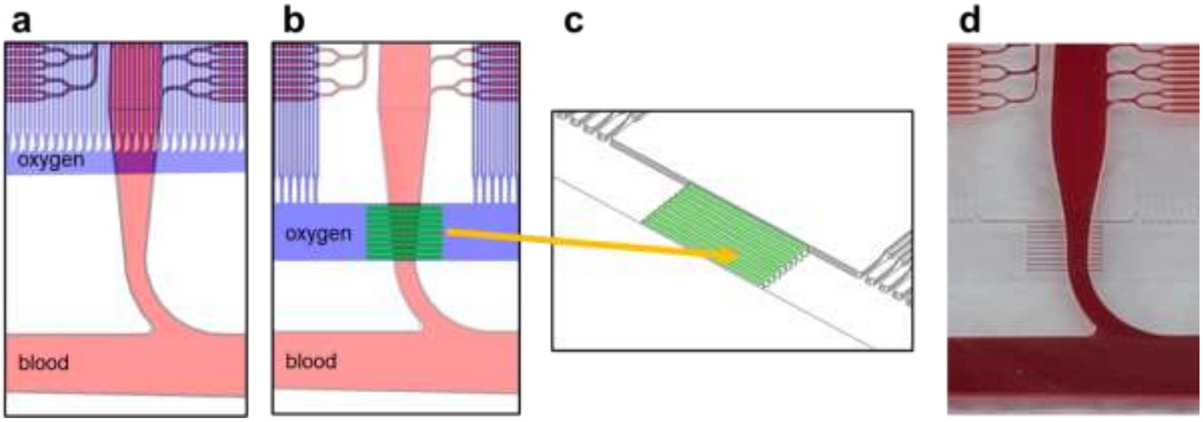

Figure S7: *Updated oxygen distribution layer to minimize overlap with blood distribution channels: a) original channel overlay with oxygen transfer channels overlapping with blood finger channels, b) updated channel overlay with no oxygen transfer channels over the blood finger channels and addition support features in the oxygen trunk channel where it overlaps with the blood finger channel, c) isometric view of additional oxygen channel support features, d) image of blood flowing through device with updated oxygen channel layer.*

1.6 Ramp comparison between BLOx and RAD2V2

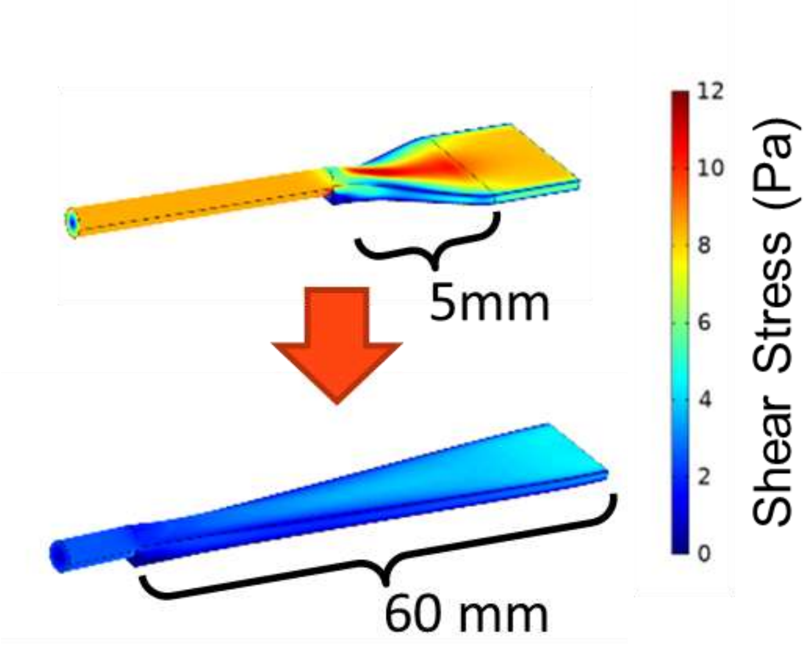

Figure S8: *Improvement in shear stress distribution in inlet transition region of the BLOx device compared to our previous design*[75] *using the iterative geometry refinement technique described in the main text.*

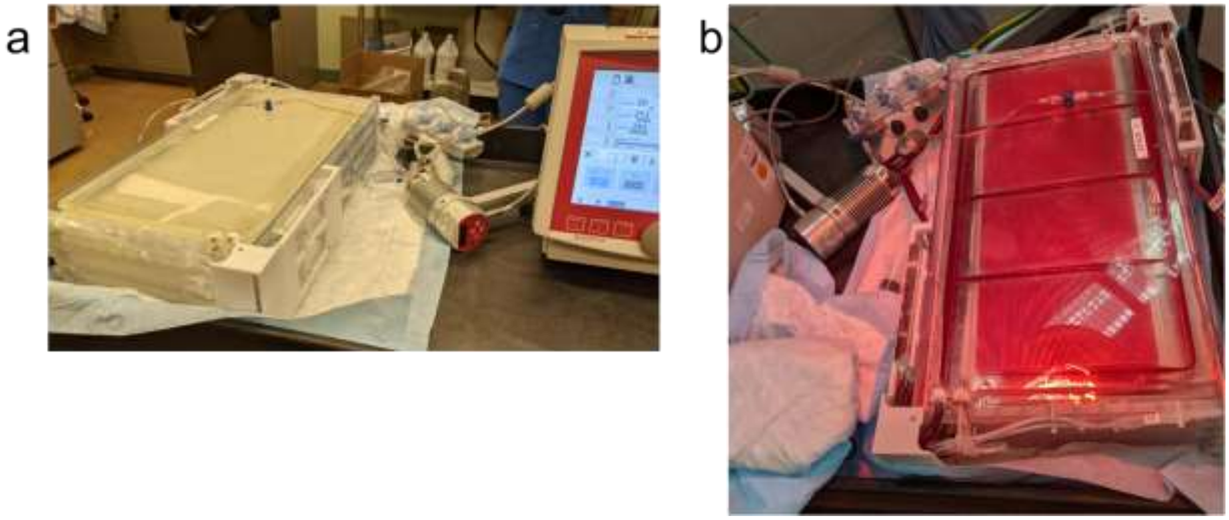

Figure S9. An 8-layer BLOx device during animal study #1 at AREVA Laboratory. a) The device primed with heparinized saline and connected to the centrifugal pump, pressure sensors and Novalung consol. b) The device fully assembled into the in vivo circuit and filled with patient blood approximately 3 hours into the study.
